# Knowledge and attitudes of adolescent girls and their mothers about early pregnancy: a cross-sectional study

**DOI:** 10.1186/s12884-022-04551-z

**Published:** 2022-03-14

**Authors:** Somayyeh Naghizadeh, Mojgan Mirghafourvand

**Affiliations:** 1 Department of Midwifery and Reproductive health, Tehran University of Medical Sciences, Tehran, Iran; 2 Department of Midwifery, Tabriz Branch, Islamic Azad University, Tabriz, Iran; 3 Social Determinants of Health Research Centre, Faculty of Nursing and Midwifery, Department of midwifery, Tabriz University of Medical Sciences, 513897977 Tabriz, Islamic Republic of Iran; 4 Department of Family Health, Social Determinants of Health Research Center, Ardabil University of Medical Sciences, Ardabil, Iran

**Keywords:** Knowledge, Attitude, Adolescent pregnancy, Adolescent girls, Mother

## Abstract

**Background:**

Adolescent pregnancy is an important public health problem and a socio-economic challenge in diverse societies. As a tremendously important problem, this issue has caused major concerns, as it exposes adolescent girls to social isolation and physical and psychological harm. So, this study aimed to determine the knowledge and attitude of adolescent girls and their mothers about early pregnancy, its causes, consequences, and predictors in Tabriz-Iran in 2020–21.

**Methods:**

This cross-sectional study was done with 540 people (270 adolescent girls and 270 mothers) in the health centers of Tabriz. Data were collected using the questionnaires of sociodemographic information, knowledge, and adolescent girls’ attitudes and their mothers about early pregnancy, its causes, and consequences. Multivariate logistic regression was used to determine the predictors of adolescent girls and their mothers’ attitudes toward early pregnancy.

**Results:**

The mean (SD: Standard Deviation) of knowledge of adolescent girls and their mothers about early pregnancy was 5.17 (3.11) and 5.57 (3.01), respectively (score range: 0 to 9). Most girls (94.1%) and mothers (87.1%) opposed pregnancy before 18. There was a statistically significant relationship between the knowledge and attitudes of girls (*p* < 0.001) and mothers (*p* < 0.001) about pregnancy at a young age. Adolescent girls and their mothers mentioned the lack of sufficient knowledge about sexual relations (57.4% of girls and 66.3% mothers agree) and the lack of knowledge about contraceptive methods (51.9% girl and 59.2% mother agree) important reasons for early pregnancy. Based on the multivariate logistic regression model and controlling for potentially confounding variables, girls whose parents were married under the age of 18 were about three times more likely to agree to early pregnancy than girls whose parents married over the age of 18 (OR = 3.10; 95% Cl: 0.90 to 10.69; *p* = 0.037). Also, mothers whose other children were married before 18 were almost five times more likely to agree to early pregnancy than women whose other children were not married before 18 (OR = 4.91; 95% Cl: 1.60 to 15.10; *p* = 0.045).

**Conclusions:**

The current study results indicate that despite the negative attitude of adolescent girls and their mothers towards early pregnancy, they had a low level of knowledge about early pregnancy. Consequently, increasing the level of knowledge of girls and their families about the consequences of marriage and pregnancy at an early age and creating a culture to correct cultural and social misconceptions to prevent marriage and pregnancy of children can reduce the severity of this damage.

**Supplementary Information:**

The online version contains supplementary material available at 10.1186/s12884-022-04551-z.

## Background

Adolescent pregnancy is defined as pregnancy in girls aged 10 to 19 years [1, 2]. It is projected that approximately 21 million girls between the ages of 15 and 19 in the developing world become pregnant each year, and approximately 12 million of them give birth [3], and at least 777,000 girls under the age of 15 give birth in these areas each year [4] which accounts for almost 11% of all births worldwide [5]. More than 90% of these births occur in low- and middle-income countries [5].

Pregnancy can be understood as a transmission and part of a woman’s transition to motherhood [6]. However, because marriages before the age of 18 are a reality for many young girls, and in many parts of the world, parents encourage their daughters to marry while they are still children, hoping that this marriage will also be economically and socially beneficial for them and at the same time reduce the financial problems of the family. However, on the other hand, early marriage endangers these girls’ physical and mental development and often leads to early pregnancy and social isolation [7].

Adolescent pregnancy is an important public health problem, and it is considered a socio-economic challenge for different societies [8]. Complications and problems related to pregnancy occur in pregnant adolescents and other pregnant women. In addition, there are more concerns for people under the age of 15, as they are not physically strong enough to maintain pregnancy and childbirth [9]. Each year, about 3.9 million girls between the ages of 15 and 19 are subjected to unsafe abortions [10]. These adolescent mothers are at higher risk than women aged 20 to 24 for eclampsia, postpartum endometritis, and systemic infections [11, 12]. Pregnancy in adolescence is a risk factor for impaired education, future unemployment, sexually transmitted infections, HIV, preterm delivery, and poor mental health [13]. Based on the United Nations, complications from pregnancy and childbirth are the leading cause of death for 15- to 19-year-old girls in developing countries. UNICEF estimates that of the 50,000 deaths, almost all occur in low- and middle-income countries [14].

Rohmah et al. (2020) in Indonesia, in their study, identified four main influential variables in adolescent pregnancy: age, economic status, level of education, and employment status [15]. The results of a study in India in 2017 showed that 59.6% of female students in the school had low knowledge about early marriage and early pregnancy, and more than half of school girls (52.5%) had a relatively favorable attitude towards early marriage and early pregnancy [6].

Because child marriage and child pregnancy are a violation of the rights of the child exposes them to serious health risks due to early pregnancy and its complications, as well as to social isolation and psychological trauma, and these negative effects of child marriage and pregnancy are not limited to themselves and spread to the family and community [16–19]. Since, according to the available literature, few studies have examined the knowledge and attitudes of adolescent girls and their mothers about pregnancy at an early age - so conducting such studies in the Iranian cultural context on adolescent girls who are exposed to this social phenomenon seems important. So, this study aimed to determine the knowledge and attitudes of adolescent girls and their mothers about early pregnancy, its causes, consequences, and predictors in Tabriz-Iran.

## Methods

### Type of study and participants

This study is a part of a large cross-sectional study that the results of another part about early marriage published previously [20]. The present research is a cross-sectional study, and it was carried out from September 2020 through March 2021 on 540 adolescent girls and their mothers in the city of Tabriz-Iran. All girls aged between 14 to 18 years and their mothers of Iranian nationality and living in Tabriz were eligible to participate in this study. Exclusion criteria were not completing the questionnaire items completely and being unwilling to continue the study. In the present study, early pregnancy was considered pregnancy under 18 years.

In order to estimate the sample size in the present research, the formula for the estimation of a ratio was employed. In the current study, the value of z with a 95% confidence level was 1.96, and the value of p (attitude towards marriage and early pregnancy) was 47.5% based on the study of Vandana et al. [6] and considering d (precision) equal to 0.08, a sample size of 150 people was obtained. Regarding cluster sampling and considering the design effect equal to 1.5 and a possible fall of 20%, the sample size was equal to 270 people. In this study, 270 adolescent girls and 270 mothers were studied.

### Sampling

After obtaining the code of ethics from the ethics committee of Tabriz University of Medical Sciences (code: IR.TBZMED.REC.1399.482) and submitting a letter of introduction to the health center of Tabriz province, the relevant permit was obtained for sampling. The research environment in the present study was health centers in Tabriz. Sampling was done based on population and cluster random sampling method because Tabriz is a large metropolis with different cultures, so in this study, we tried to give all the female adolescents of this city a chance to be selected. The sampling method was that first, the list of 83 health centers in the ten districts of Tabriz was determined, and 18 health centers were randomly selected from them. Then, the determined sample size was divided among the selected health centers proportionally. Families with 14–18-year-old girls who met the inclusion criteria were identified through the SIB system (Integrated Health System) in the mentioned centers, and a number of these families were randomly selected. Then during a telephone call with these people, the researcher introduced herself at the beginning conversation explained the aims of the study. If the mother and daughter agree to participate in the study, they were asked to come to the relevant health center on a specific date to be given the questionnaires to complete them, or if the participants wished and consented, an anonymous e-mail or anonymous mobile number was received from them. The questionnaire link was sent to them to fill in at home stress-free and confidential. It must be noted that the SIB system has covered more than 90–92% of the population living in this city. Before starting the study, participants were given the necessary explanations about the objectives and method of the study, voluntary participation, privacy, confidentiality, and the right to refrain from continuing in all stages of data collection. They were assured that there was no need to write their name and surname and that all their information would remain confidential with the researcher, and informed written consent was obtained from them.

### Data collection tools

In the current study, two researcher-made questionnaires were used to collect data: the questionnaire of knowledge and attitude of adolescent girls about early pregnancy, its causes, and consequences, and the questionnaire of knowledge and attitude of mothers of adolescent girls about early pregnancy, its causes, and consequences.

#### The girls’ questionnaire had four parts

Sociodemographic information questionnaire for adolescent girls: including age, marital status, number of sister(s) or brother(s), economic status, being the child of divorce. Some other characteristics including parental marriage, siblings or close relatives under the age of 18, traditional parental marriage, appropriate age of girls’ marriage, the final decision–maker on whether to marry girls, and how to oppose your parents if they decide to marry girls before the age of 18 were also assessed.

The Adolescent Girls Knowledge Questionnaire about early pregnancy had nine questions with “right, wrong, and I do not know” answers. Adolescent girls’ attitudes were measured on a 5-point Likert scale (totally agree, agree, no opinion, disagree, totally disagree) using the item “I would like to have children before 18”. The adolescent girls’ attitude questionnaire on the causes and consequences of early pregnancy had eight questions and was measured based on the 5-option Likert scale (totally agree, agree, no opinion, disagree, totally disagree).

#### The mothers’ questionnaire had four parts

Sociodemographic information questionnaire for mothers included questions about age, marriage age, number of girl(s) and boy(s), marital status, job, education, job, and spouse’s education. Some other characteristics including the marriage of your other children under the age of 18 years old, appropriate age of girls’ marriage, the final decision–maker on your daughter’s marriage and disagreement with your daughter if she decides to marriage under the age of 18 years old were also assessed.

The Mothers Knowledge Questionnaire about early pregnancy had nine questions with “right, wrong, and I do not know” answers. Mothers’ attitudes were measured on a 5-point Likert scale (totally agree, agree, no opinion, disagree, totally disagree) using the item “ You would like your daughter to have children before the age of 18?”. The mothers’ attitude questionnaire on the causes and consequences of early pregnancy had eight questions and was measured based on the 5-option Likert scale (totally agree, agree, no opinion, disagree, totally disagree).

The validity and reliability of the current questionnaires were assessed before sampling. Qualitative and quantitative methods were used to evaluate the content validity. To evaluate the validity of qualitative content, the questionnaire was reviewed by ten experts in this field. These people included Midwifery and Reproductive Health of Tabriz and Tehran Universities of Medical Sciences faculty members. After receiving comments, the necessary corrections were made. In the quantitative method, content validity was assessed using content validity ratio (CVR) and content validity index (CVI). The results of quantitative content validity also revealed that CVR and CVI values ​​are 0.88 and 0.84, respectively. The reliability of the present questionnaires was also obtained using the internal consistency method with Cronbach’s alpha coefficient. This coefficient was 0.85 for “girls’ knowledge questionnaire,” for “girls’ attitudes on the causes and consequences of early pregnancy” was 0.68, for “mothers’ knowledge” was 0.87 and for “mothers’ attitudes on the causes and consequences of early pregnancy” was 0.69. The knowledge assessment tool is available as Additional file 1.

### Statistical analysis

The data were analyzed using SPSS, 21. The normality of the data was determined by Kurtosis and Skewness, all of which had a normal distribution. Descriptive statistics, including frequency (percentage), mean and standard deviation (SD), were used to report the knowledge and attitude of adolescent girls and their mothers and the causes and consequences of early pregnancy. One-way analysis of variance in the bivariate analysis was used to investigate the relationship between knowledge score and attitude of adolescent girls and their mothers about early pregnancy. A chi-square test was used to assess the relationship between sociodemographic characteristics and attitude (The options “I completely agree” and “I agree” were merged and the options “I have no opinion,” “I disagree”, and “I completely disagree” were merged and the attitude variable was transformed to a two-state qualitative variable). Then, variables with *p* < 0.05 were entered into a multivariate logistic regression to control confounding factors. The results of multivariate logistic regression were reported as adjusted odds ratio (aOR) with a 95% confidence interval (95% CI). *P* < 0.05 was considered statistically significant.

## Results

The mean (SD) of girls’ age was 16.63 (1.16) years. Only 8 (3%) of the girls were children of divorce. Nearly one-fifth of girls (19.6%) said their parents were married before 18. More than half of the girls (59.6%) considered the appropriate age for marriage to be 21–25 years. About two-thirds of girls (67%) said they would make the final decision on their marriage, and the majority (83.3%) said they would oppose it if their parents decided to marry before the age of 18 (Table 1).

**Table 1:** Characteristics of adolescent girls and their relationship with attitude about early pregnancy (*n* = 270)

Characteristic	Number (%)	Agreement with child marriage Number (%)	*P*-value*	Characteristic	Number (%)	Agreement with child marriage Number (%)	*P*-value*
**Age (Year)**			0.822	**Appropriate age of marriage for girls**			0.001
14	12 (4.4)	1 (6.3)		16–20	26 (9.6)	7 (43.8)	
15	39 (14.4)	3 (18.8)		21–25	161 (59.6)	6 (37.5)	
16	64 (23.7)	3 (18.8)		26≤	83 (30.7)	3 (18.7)	
17	78 (28.9)	6 (37.5)		**Child of divorce**			0.391
18	77 (28.5)	3 (18.8)		Yes	8 (3.0)	1 (6.3)	
**Number of sisters**			0.080	No	262 (97.0)	15 (93.7)	
0	136 (50.4)	7 (43.8)		**The traditional marriage of parents**			0.367
1	109 (40.4)	5 (31.3)		Yes	207 (76.9)	10 (62.5)	
≥ 2	25 (9.3)	4 (25.0)		No	63 (23.3)	6 (37.5)	
**Number of brothers**			0.783	**The marriage of parents under 18 years of age**			0.042
0	116 (43.0)	7 (43.8)		Yes	53 (19.6)	7 (43.8)	
1	132 (48.9)	7 (43.8)		No	217 (80.4)	9 (56.2)	
≥ 2	22 (8.1)	2 (12.5)		**The marriage of siblings under 18 years of age**			0.903
**Economic status**			0.606	Yes	15 (5.6)	1 (6.3)	
Poor	17 (6.3)	2 (12.5)		No	255 (94.4)	15 (93.7)	
Moderate	142 (52.6)	9 (56.2)		**The marriage of close relatives under 18 years of age**			
Good	111 (41.1)	5 (31.3)		Yes	176 (65.2)	11 (68.8)	0.042
**Education**			0.516	No	94 (34.8)	5 (31.2)	
9th grade	42 (14.1)	3 (18.8)		**Final decision-maker about the girls’ marriage**			0.038
10th grade	67 (22.3)	5 (31.3)		My self	181 (67.0)	6 (37.5)	
11th grade	79 (26.3)	2 (12.5)		Father or mother	89 (33.0)	10 (62.5)	
12th grade	112 (37.3)	6 (37.6)		**Disagreement with parents if they decide to girls’ marriage under the age of 18 years old**			
**Marital status**			0.601	Yes	225 (83.3)	8 (50.0)	0.001
Single	261 (96.7)	15 (93.8)		No	45 (16.6)	8 (50.0)	
Married	9 (3.3)	1 (6.2)					

* Chi-square test

The mean (SD) of mothers’ age was 41.35 (4.16) years. The mean (SD) age of mothers’ marriage was 20.52 (4.14) years. One-third of them (39.3%) had a university degree, and about a quarter of them (73.7%) were housewives. Nearly two-thirds of them considered the appropriate age for marriage for girls to be 21–25 years old. 37 (13.8%) mothers stated that their other children were married before 18. About three-quarters of mothers (71.1%) said they would oppose their daughter if they decided to marry before 18 (Table 2).

**Table 2:** Characteristics of mothers and their relationship with attitude about early pregnancy (*n* = 270)

Characteristic	Number (%)	Agreement with child marriage Number (%)	***P***-value*	Characteristic	Number (%)	Agreement with child marriage Number (%)	***P***-value*
**Age** (Year)			0.501	**marriage age**			0.435
< 40	134 (49.7)	17 (48.6)		< 20	121 (44.8)	19 (54.3)	
41–50	114 (42.2)	13 (37.1)		≥20	149 (55.1)	16 (45.7)	
> 50	22 (8.1)	5 (14.3)		**Number of girls**			0.017
**Education**			0.001	1	162 (60.0)	22 (62.9)	
Illiterate/Elementary school	40 (14.9)	14 (40.0)		2	84 (31.1)	6 (17.1)	
Secondary school	35 (13)	5 (14.3)		≤3	24 (8.9)	7 (20.0)	
High school/diploma	106 (39.3)	10 (28.6)		**Number of boys**			0.001
University	89 (33.0)	6 (17.1)		0	109 (40.4)	10 (29.4)	
**Job**			0.023	1	125 (46.3)	9 (26.5)	
Housewife	199 (73.7)	32 (91.4)		≥2	36 (13.3)	15 (44.1)	
Working at home and outside the home	72 (26.3)	2 (5.7)		**The marriage of other children under 18 years of age**			
**Spouse Education**			0.116	Yes	37 (13.8)	16 (45.7)	0.001
Illiterate/Elementary school	38 (14.1)	7 (20.6)		No	233 (86.2)	19 (54.3)	
Secondary school	47 (17.4)	7 (20.6)		**Appropriate age of marriage for girls**			
High school diploma	92 (34.1)	12 (35.3)		16–20	43 (15.9)	15 (42.9)	0.001
University	93 (34.4)	8 (23.5)		21–25	165 (61.1)	16 (45.7)	
**Spouse Job**			0.945	≥26	62 (23.0)	4 (11.4)	
Unemployed	12 (4.5)	1 (2.9)		**Final decision-maker about your daughter’s marriage**			
Freelance	126 (46.7)	17 (50.0)		My daughter	146 (54.1)	12 (34.3)	0.021
Employee	88 (32.6)	10 (29.4)		Her father and me	124 (45.9)	23 (65.7)	
Laborer	44 (16.3)	6 (17.6)		**Disagreement with your daughter if she decides to marriage under the age of 18 years old**			
**Marital status**			0.030	Yes	192 (71.1)	16 (45.7)	0.001
Married	255 (94.4)	30 (85.7)		No	79 (28.9)	19 (54.3)	
Widowed/ Divorced	15 (5.6)	5 (14.3)					

* Chi-square test

The mean (SD) of adolescent girls and their mothers’ knowledge about early pregnancy was 5.17 (3.11) and 5.57 (3.01) from the score range of 0–9, respectively. In the case of girls, 112 (41.5%) had good knowledge, 74 (27.4%) had moderate knowledge, and 84 (31.1%) had poor knowledge about early pregnancy, and in the case of mothers, 122 (45.2%) had good knowledge, 80 (29.6%) had moderate knowledge, and 68 (25.2%) had poor knowledge about early pregnancy. The study of the relationship between girls’ and mothers’ knowledge about early pregnancy showed no statistically significant relationship between the two groups (*p* < 0.539).

Investigating adolescent girls’ attitudes about early pregnancy showed that only 3 (1.1%) agreed with pregnancy before the age of 18, 13 (4.8%) had no opinion, and the majority of girls, 254 (94.1%), opposed to pregnancy before 18, and of their mothers, only 12 (4.4%) agreed with pregnancy before the age of 18, 23 (8.5%) had no opinion, and the majority of mothers, 235 (87.1%) were against pregnancy before the age of 18. There was a statistically significant relationship between girls’ and their mothers’ attitudes about early pregnancy (*p* < 0.001).

Also, the study of the relationship between knowledge and attitudes of participants revealed that there was a statistically significant relationship between knowledge and attitudes of girls (*p* < 0.001) and mothers (*p* < 0.001) about early pregnancy (Table 3).

**Table 3:** Relationship between knowledge and attitude of adolescent girls and mothers about early pregnancy

Variable	adolescent girls Number (%)		Mother Number (%)		The relationship between knowledge and attitude of mothers and girls (***P***-value)
**Knowledge regarding early pregnancy (Score range)**					0.539*
Good (Score: 7–9)	112 (41.5)		122 (45.2)		
Average (Score: 4–6)	74 (27.4)		80 (29.6)		
Poor (Score: 0–3)	84 (31.1)		68 (25.2)		
**Attitude regarding early pregnancy**	**Number (%)**	**Mean (SD) of knowledge**	**Number (%)**	**Mean (SD) of knowledge**	< 0.001*
Agree	3 (1.1)	5.37 (3.02)	12 (4.4)	5.94 (2.70)	
No opinion	13 (4.8)	1.54 (2.47)	23 (8.5)	3.00 (3.43)	
Disagree	254 (94.1)	3.67 (4.72)	235 (87.1)	3.10 (3.82)	
**The relationship between knowledge and attitude**	*P* < 0.001**		*P* < 0.001**		

* Chi-square test; ** One-way ANOVA

The study of attitudes about the causes and consequences of pregnancy at a young age indicated that adolescent girls mentioned the most important reasons for early pregnancy as lack of sufficient knowledge about sex (57.4% agree and totally agree), lack of knowledge about contraceptive methods (51.9% agree totally agree), and pressure from husband and husband’s family (50.4% agree totally agree) and 67.8% of girls disagreed that “pregnancy in girls under 18 is no different from people over 18”. About 71% of adolescent girls believed that they could not meet the educational needs of their children because girls under the age of 18 were growing mentally, and 62.2% of girls believed that they could not meet the physical development and the nutritional needs of their fetus because girls under the age of 18 are growing physically (Table 4).

**Table 4:** Adolescent girls’ attitude about the causes and consequences of early pregnancy

Variable	Totally agree	Agree	No opinion	Disagree	Totally disagree
1. The cause of pregnancy before the age of 18 is the desire and insistence of the girl’s family.	2 (0.7)	1 (0.4)	17 (6.3)	24 (8.9)	226 (83.7)
2. The cause of pregnancy before the age of 18 is the pressure of the husbands and the husband’s family.	42 (15.6)	94 (34.8)	97 (35.9)	15 (5.6)	22 (8.1)
3. The cause of pregnancy before the age of 18 is the lack of knowledge about the use of contraceptive methods.	55 (20.4)	85 (31.5)	90 (33.3)	15 (5.6)	25 (9.3)
4. The cause of pregnancy before the age of 18 is fear of subsequent infertility.	19 (7.0)	57 (21.1)	121 (44.8)	42 (15.6)	31 (11.5)
5. The cause of pregnancy in girls under 18 is not having enough knowledge about sex.	53 (19.6)	102 (37.8)	91 (33.7)	12 (4.4)	12 (4.4)
6. Pregnancy in girls under 18 is no different from people over 18.	9 (3.3)	22 (8.1)	56 (20.7)	70 (25.9)	113 (41.9)
7. Because girls under the age of 18 are physically growing, they cannot meet the nutritional needs of their fetuses and babies.	70 (25.9)	98 (36.3)	79 (29.3)	17 (6.3)	6 (2.2)
8. Because girls under the age of 18 are developing intellectually, they cannot meet their children’s educational needs.	99 (36.7)	93 (34.4)	53 (19.6)	16 (5.9)	9 (3.3)

The mothers of these girls mentioned the most important reasons for early pregnancy as lack of knowledge about sexual relations (66.3% agree and totally agree), lack of knowledge about contraceptive methods (59.2% agree and totally agree), and pressure from the husband and husband’s family (52.2% agree and totally agree) and 65.2% of mothers were against the statement that “Pregnancy in girls under 18 is no different from people over 18.” Almost 68% of mothers believed that they could not meet the educational needs of their children because their girls were under 18 years of age, and 64.1% of them believed that girls under the age of 18 could not meet the physical development and nutritional needs of their fetus because they are growing physically (Table 5).

**Table 5:** Mothers attitude about the causes and consequences of early pregnancy

Variable	Totally agree	Agree	No opinion	Disagree	Totally disagree
1. The cause of pregnancy before the age of 18 is the desire and insistence of the girl’s family.	8 (3.0)	9 (3.3)	26 (10.7)	61 (22.6)	163 (60.4)
2. The cause of pregnancy before the age of 18 is the pressure of the husbands and the husband’s family.	48 (17.8)	93 (34.4)	91 (33.7)	26 (9.6)	12 (4.4)
3. The cause of pregnancy before the age of 18 is the lack of knowledge about the use of contraceptive methods.	73 (27)	87 (32.2)	69 (25.6)	25 (9.3)	16 (5.9)
4. The cause of pregnancy before 18 is fear of subsequent infertility.	32 (11.9)	79 (29.3)	89 (33.0)	47 (17.4)	23 (8.5)
5. The cause of pregnancy in girls under 18 is not having enough knowledge about sex.	57 (21.1)	122 (45.2)	64 (23.7)	19 (7.0)	8 (3.0)
6. Pregnancy in girls under 18 is no different from people over 18.	8 (3)	23 (8.5)	63 (23.3)	91 (33.7)	85 (31.5)
7. Because girls under the age of 18 are physically growing, they cannot meet the nutritional needs of their fetuses and babies.	81 (30.0)	92 (34.1)	58 (21.5)	29 (10.7)	10 (3.7)
8. Because girls under the age of 18 are developing intellectually, they cannot meet their children’s educational needs.	82 (30.4)	105 (38.9)	54 (20.0)	22 (8.1)	7 (2.6)

Studying the relationship between sociodemographic characteristics and adolescent girls’ attitudes about early pregnancy using chi-square test showed there was a statistically significant relationship between girls’ attitude and variables of the appropriate age for marriage (*p* = 0.001), the marriage of parents under 18 years of age (*p* = 0.042), the marriage of distant or close relatives under 18 years of age (*p* = 0.042), the final decision maker about girls marriage (*p* = 0.038) and opposition to parents if they decided to marry girls before the age of 18 (*p* = 0.001). These variables, along with the knowledge of adolescent girls, were included in the multivariate logistic regression model. The results by controlling socio-demographic characteristics as possible confounding variables showed that girls whose parents were married under the age of 18 were about three times more likely to agree to early pregnancy than girls whose parents married over the age of 18 (OR = 3.10; 95% Cl: 0.90 to 10.69; *p* = 0.037) (Table 6).

**Table 6:** Predictors of adolescent girls’ attitude about early pregnancy based multivariable regression logistic model

Variable	aOR (95% CI)^**a**^	***P***-value
**Knowledge** (Reference: Poor)	–	–
Good	1.62 (0.45 to 5.82)	0.462
Average	0.65 (0.13 to 3.14)	0.589
**Appropriate age of marriage for girls** (Reference: ≥26)	–	–
16–20	4.04 (0.71 to 23.09)	0.117
21–25	0.59 (0.13 to 2.87)	0.508
**The marriage of parents under the age of 18 years old** (Reference: No)	–	–
Yes	3.53 (1.01 to 12.42)	0.049
**The marriage of close relatives under the age of 18 years old** (Reference: No)	–	–
Yes	0.46 (0.12 to 1.71)	0.247
**Final decision-maker about the girls’ marriage** (Reference: Father or mother)	–	–
Myself	0.20 (0.03 to 1.43)	0.109
**Disagreement with parents if they decide to girls’ marriage under the age of 18 years old** (Reference: No)	–	–
Yes	0.36 (0.10 to 1.32)	0.124

^a^ Adjusted Odds Ratio (95% Confidence Interval)

Examining the relationship between sociodemographic characteristics and mothers’ attitudes about early pregnancy based on chi-square test showed there was a statistically significant relationship between mothers’ attitudes and the variables of mother’s education (*p* = 0.001), mother’s job (*p* = 0.023), marital status (*p* = 0.030), number of daughters (*p* = 0.017), number of sons (*p* = 0.001), the marriage of other children under 18 years of age (*p* = 0.001), appropriate age for marriage (*p* = 0.001), being the final decision maker about daughter’s marriage (*p* = 0.021) and opposition to a daughter if she decided to marry before the age of 18 with mothers’ attitudes (*p* = 0.001). These variables were included in the multivariate logistic regression model and the maternal knowledge variable. The results by controlling socio-demographic characteristics as possible confounding variables showed that women whose other children were married under 18 were more likely to agree to early pregnancy. It was almost five times higher than women whose other children were not married before the age of 18 (OR = 4.91; 95% Cl: 1.60 to 15.10; *p* = 0.045) (Table 7).

**Table 7:** Predictors of mothers’ attitude about early pregnancy based multivariable regression logistic model

Variable	aOR (95% CI)^**a**^	***P***
**Knowledge** (Reference: Poor)	–	–
Good	2.13 (0.72 to 6.33)	0.171
Average	0.48 (0.12 to 1.84)	0.286
**Mother’s Education **(Reference: University)	–	–
Illiterate/Elementary school	0.75 (0.14 to 4.01)	0.735
Secondary school	0.79 (0.16 to 3.74)	0.767
High school/diploma	0.65 (0.18 to 2.25)	0.496
**Job **(Reference: Working at home or outside the home)	–	–
Housewife	3.89 (0.90 to 16.75)	0.068
**Marital status **(Reference: Widowed/ divorced)	–	–
Married	0.73 (0.14 to 3.63)	0.700
**Number of girls **(Reference: ≥3)	–	–
1	1.56 (0.24 to 10.00)	0.639
2	0.38 (0.05 to 2.79)	0.339
**Having a son **(Reference: No)	–	–
Yes	1.42 (0.52 to 3.85)	0.486
**Appropriate age of marriage for girls** (Reference: ≥26)	–	–
16–20	2.51 (0.56 to 11.22)	0.226
21–25	1.07 (0.30 to 3.77)	0.917
**The marriage your other children under the age of 18 years old** (Reference: No)	–	–
Yes	4.91 (1.60 to 15.10)	0.045
**Final decision-maker about your daughter’s marriage **(Reference: Her father and me)	–	–
My daughter	0.33 (0.07 to 1.50)	0.153
**Disagreement with your daughter if she decides to marriage under the age of 18 years old** (Reference: No)	–	–
Yes	2.89 (0.59 to 14.21)	0.191

^a^ Adjusted Odds Ratio (95% Confidence Interval)

## Discussion

The study results showed that only about 40% of adolescent girls and their mothers had good knowledge about early pregnancy. The majority of girls and their mothers were against pregnancy before 18. There was a statistically significant relationship between knowledge and attitude of girls and mothers about early pregnancy. Adolescent girls and their mothers cited the lack of sufficient knowledge about sexual relations, contraceptive methods, and pressure from husband and husband’s family as the most important reasons for early pregnancy. Based on the multivariate logistic regression model and controlling for potentially confounding variables, girls whose parents were married under the age of 18 were about three times more likely to agree to pregnancy at a younger age than girls whose parents married over the age of 18 and mothers whose other children were married before the age of 18 were almost five times more likely to agree to pregnancy at a younger age than women whose other children were not married before the age of 18.

The research results revealed that about 40% of girls had good knowledge about early pregnancy, but the majority of girls were against pregnancy before the age of 18, and more than half of schoolgirls had a relatively favorable attitude, and nearly half of the girls had a favorable attitude towards early marriage [6]. The results of this study were not consistent with our study; these differences may be due to regional and cultural differences. It should be noted that no study examined the knowledge and attitudes of mothers.

Adolescent girls and their mothers mentioned a lack of sufficient knowledge about sexual relations, lack of knowledge about contraceptive methods, and pressure from husbands and their families as the most important reasons for pregnancy at a young age. In Osaikhuwuomwan et al.’s study, the most important reasons for pregnancy in adolescents were peer pressure to have sex (71.8%), having sex without considering the consequences such as pregnancy (60.1%), having unprotected sex (60.1%), and boys or men refusing to use condoms (52.1%). In this study, 10.4% of people believed that they could get pregnant to get married [8]. It can be said that in both studies, lack of sufficient knowledge about sex and not using contraceptive methods are the reasons for adolescent pregnancy, but what seems to be important here is that due to the nature and cultural and social values of Iranian society, marriage is a prerequisite for girls in Iran, which conflicts with the study of Osaikhuwuomwan. In Ayanaw Habitu et al.’s study, 65% of girls mentioned marriage as the reason for pregnancy [6], which did not agree with the present study results. Likewise, in Iranian culture, the role of the family in the marriage and pregnancy of girls is very important [8]; in our study, girls mentioned that one of the most important reasons for adolescent pregnancy was the pressure of the husband and the husband’s family.

Since the current study results revealed that there is a statistically significant relationship between the knowledge and attitudes of girls and their mothers about early pregnancy, it is recommended that appropriate education be provided at different levels of society to increase the knowledge of girls, parents, and society. Educational programs to empower girls can be an effective measure to prevent pregnancy at a young age.

Based on the multivariate logistic regression model, girls whose parents were married under 18 and mothers whose other children were married under the age of 18 were more likely to agree to early pregnancy. Consequently, based on the results of the current study, it can be said that the recurrence of marriage and pregnancy at an early age will be higher in families in which this has happened before. So, a comprehensive approach is needed to address the issue of adolescent pregnancy. This means that instead of focusing on changing girls’ behavior, the root causes of adolescent pregnancy, such as poverty, gender inequality, social pressures, and coercion, should be addressed. This approach is possible by preventing child marriage, sexual violence, and coercion, creating gender-equitable societies by empowering girls, involving men and boys in this process, and ensuring that adolescents have access to sexual and reproductive health information.

One of the limitations of the current study is that it is cross-sectional, and the relationship between knowledge and attitude cannot be attributed to a causal relationship. There was also the possibility of response bias due to the nature of the research questions, and by assuring the participants about the confidentiality of the information and completing questionnaires without first and last name, an attempt was made to remove this restriction. Similarly, this study was conducted in Tabriz with Turkish ethnicity, so the results cannot be generalized to other cities and ethnicities in Iran [1].

## Conclusion

The current study results indicate that despite the negative attitude of adolescent girls and their mothers towards early pregnancy, they had a low level of knowledge about young pregnancy. Consequently, increasing the level of knowledge of girls and their families about the consequences of marriage and pregnancy at an early age and creating a culture to correct cultural and social misconceptions to prevent marriage and pregnancy of children can reduce the severity of this damage.

## Supplementary Information

**Additional file 1.**

## Data Availability

Datasets used and analyzed during this study are available from the corresponding author on reasonable request.

## Abbreviations

UNICEF: United Nations International Children’s Emergency Fund
95% CI: 95% Confidence Interval
SD: Standard Deviation
GLM: General Linear Model
