# Supplementary material for: Knowledge and attitudes of adolescent girls and their mothers about early pregnancy: a cross-sectional study
Source: BMC Pregnancy Childbirth. 2022 Mar 14;22:205. doi: 10.1186/s12884-022-04551-z (PMC8919530; doi:10.1186/s12884-022-04551-z)
Supplement: Supplementary file 1 — Additional file 1. [file 12884_2022_4551_MOESM1_ESM.docx]

**Knowledge of adolescent girls and their mothers about early pregnancy questionnaire**

| **Items** | **Correct** | **Wrong** | **I do not know** |
| --- | --- | --- | --- |
| 1- Early pregnancy causes unwanted pregnancy. |  |  |  |
| 2- Early pregnancy increases abortion. |  |  |  |
| 3- Early marriage reduces the distance between pregnancies. |  |  |  |
| 4- Early marriage increases the number of pregnancies. |  |  |  |
| 5- Pregnancy at the age of less than 18 years causes complications of pregnancy and childbirth. (Increased blood pressure, bleeding and infection and …) |  |  |  |
| 6- Pregnancy at the age of less than 18 years (due to pregnancy and childbirth complications) increases adolescent mothers' death. |  |  |  |
| 7- Childbirth before the age of 18 will increase the probability of death of infants and children under five years old. |  |  |  |
| 8- Pregnancy and childbirth before the age of 18 increase mental disorders such as postpartum depression. |  |  |  |
| 9- Pregnancy and childbirth before the age of 18 cause calcium and iron deficiency in adolescent girls. |  |  |  |
